# The Concerted Action of Mitochondrial Dynamics and Positioning: New Characters in Cancer Onset and Progression

**DOI:** 10.3389/fonc.2017.00102

**Published:** 2017-05-22

**Authors:** Diana Pendin, Riccardo Filadi, Paola Pizzo

**Affiliations:** ^1^Department of Biomedical Sciences, University of Padova, Padova, Italy; ^2^Neuroscience Institute, National Research Council (CNR), Padova, Italy

**Keywords:** mitochondria, cancer, mitochondrial shape, fusion, fission

## Abstract

Mitochondria are dynamic organelles whose morphology and activity are extremely variable, depending on the metabolic state of the cell. In particular, their shape and movements within the cell are finely regulated by an increasing number of proteins, which take part in the process of mitochondrial fission/fusion and connect the organelles to the cytoskeleton. As to their activities, mitochondria are considered to be at the crossroad between cell life and death since, on the one hand, they are essential in ATP production and in multiple metabolic pathways but, on the other, they are involved in the intrinsic apoptotic cascade, triggered by different stress conditions. Importantly, the process of mitochondrial Ca^2+^ uptake, as well as the morphology and the dynamics of these organelles, is known to deeply impact on both pro-survival and pro-death mitochondrial activities. Recently, increasing evidence has accrued on a central role of deregulated mitochondrial functionalities in the onset and progression of different pathologies, ranging from neurodegenerative diseases to cancer. In this contribution, we will present the latest findings connecting alterations in the machineries that control mitochondrial dynamics and localization to specific cancer hallmarks, highlighting the importance of mitochondria for the viability of cancer cells and discussing their role as promising targets for the development of novel anticancer therapies.

## Introduction

Mitochondria are organelles that orchestrate a plethora of fundamental cellular functions, ranging from ATP production, control of substrate utilization, biosynthesis of macromolecules, redox, and intracellular calcium (Ca^2+^) homeostasis. The complexity in function is reflected on an elaborated and dynamic shape, which undergoes dramatic changes during different phases of cell life and death. Mitochondrial morphology is heterogeneous among cell types and a growing body of evidence indicates that it plays a critical role in overall cell physiology. Indeed, changes in mitochondrial shape have been associated with complex processes such as development, cell division, apoptotic cell death, and neurodegeneration. Mitochondria are also dynamic as to their position within the cell and move continuously along cytoskeleton. In polarized cells, such as neurons, mitochondrial movement has been suggested to be essential for a proper subcellular distribution toward high-energy demanding regions, such as synapses (1). On the other hand, a much more ordered and stationary positioning of mitochondria has been observed in adult cardiomyocytes (2), with a “kissing/nanotubuling”-based intermitochondrial communication responsible for the sharing of material between otherwise isolated mitochondria (3). Importantly, increasing evidence has reported that mitochondrial dynamics, in terms of fission and fusion and also subcellular spatial organization, are deregulated in cancer.

**Figure 1:**
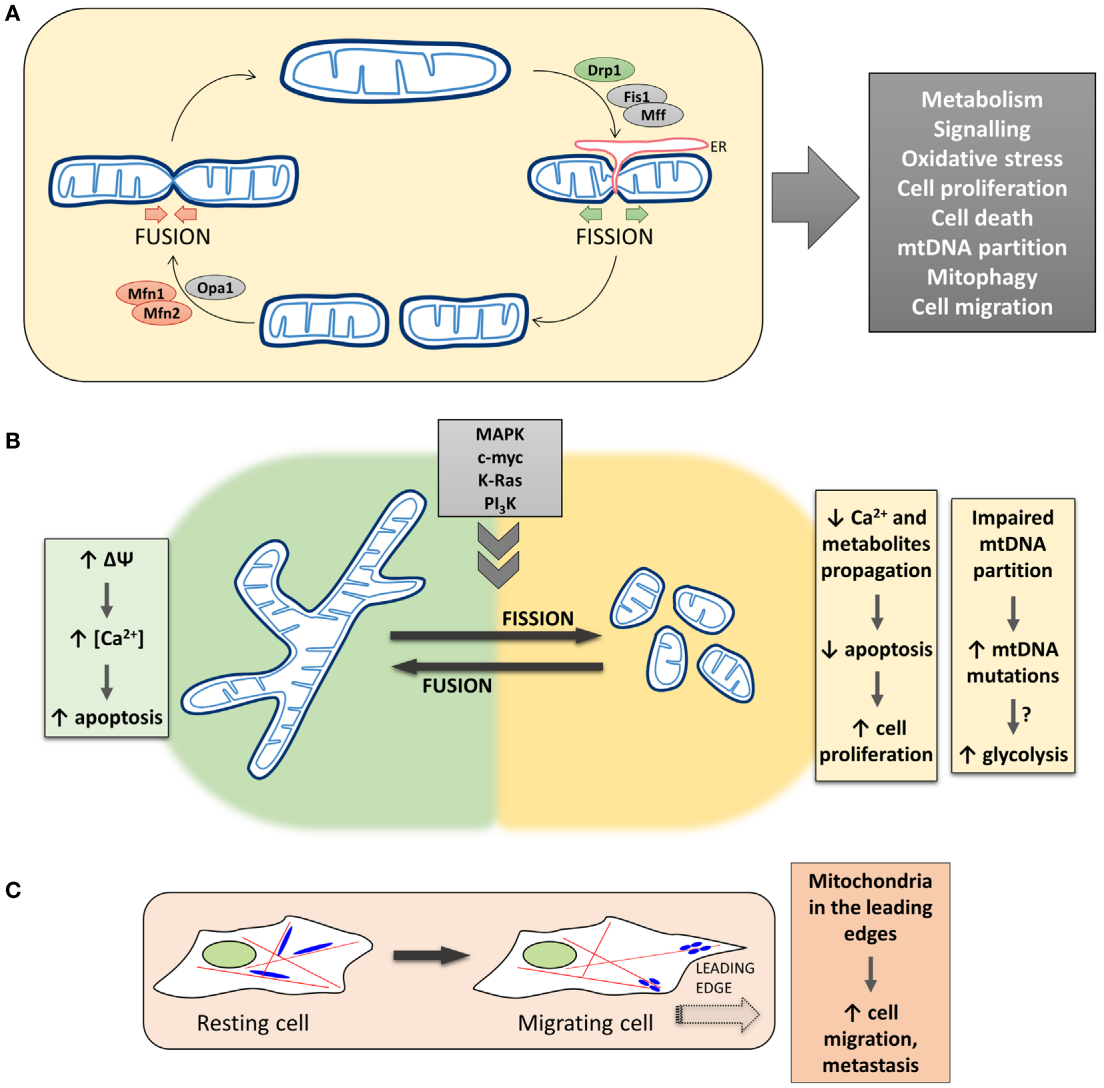
**The complex and multifaceted relationship between mitochondrial dynamics/positioning and cancer development**. **(A)** The morphology of the mitochondrial network in living cells is the result of the balance between fusion and fission events. The mitochondrial fusion machinery relies on the activity of Mfns and Opa1, while mitochondrial fission is driven by Drp1 in a regulated process that involves other proteins, such as Fis1 and Mff, and endoplasmic reticulum (ER) tubules circumscribing mitochondria. Mitochondrial morphology plays a critical role in overall cell physiology, and changes in mitochondrial shape have been associated with alterations of key physiological processes, such as cell metabolism, proliferation, and migration. **(B)** *Left*, Mitochondria fusion promotes the diffusion of metabolites throughout the entire network, sustaining mitochondrial membrane potential, respiration, and metabolism. The propagation of Ca^2+^ waves is also promoted, resulting in a Ca^2+^-dependent apoptosis induction in cancer cells. *Right*, Mitochondria fragmentation causes an impairment in Ca^2+^ diffusion within the network, which results in an increased resistance of tumor cells to apoptosis, favoring cell proliferation. Lack of mitochondrial fusion also leads to an impaired mtDNA partitioning and accumulation of mtDNA mutations, which could result in mitochondrial metabolic dysfunction. **(C)** Mitochondrial fission/fusion dynamics have also been reported to impact on the translocation of mitochondria along cytoskeleton to the leading edge of migrating cells. The presence of mitochondria in the cell periphery correlates with the metastatic potential of cancers.

The morphology of the mitochondrial network in living cells is the result of the balance between fusion and fission events (Figure 1A). The mitochondrial fusion machinery relies on the GTPases mitofusin 1 (Mfn1) and mitofusin 2 (Mfn2), located in the outer mitochondrial membrane (OMM) (4), and optic atrophy 1 (Opa1), located in the inner mitochondrial membrane (IMM) (5). On the other side, mitochondrial fission is driven by the cytosolic GTPase dynamin 1-like protein (DNM1L/Drp1) (6), that is recruited to the OMM in a regulated process, that involves also other fission-related proteins such as fission 1 (Fis1) (7), mitochondrial fission factor (Mff) (8), MiD51, and MiD49 (9). Recently, a role for endoplasmic reticulum (ER) in determining the sites at which fission will occur has been proposed, suggesting that, before the recruitment of Drp1 to the OMM, mitochondria are constricted at points of contact with the ER (10). In particular, ER tubules circumscribe mitochondria, followed by Drp1 recruitment and oligomerization to form a ring-like structure that wraps and divides mitochondria, fueled by Drp1 GTPase activity. Actin polymerization, modulated by the ER protein inverted formin-2, is also involved in ER-mediated mitochondrial division (11).

A proper mitochondrial distribution is also essential for cell functionality, especially in polarized cells, such as neurons. Anterograde (away from cell body) and retrograde transport of mitochondria along microtubules are mediated by plus-end-directed kinesin-1/KIF5 and minus-end-directed dynein motor proteins, respectively (12, 13). The interaction between mitochondria and kinesin/dynein is allowed by the formation of a complex between the OMM protein Miro1 and Milton (whose mammalian homologs are TRAK1/2), that in turn interacts with the motors (14–16). Importantly, Miro1 is endowed with EF-hand domains, essential for Ca^2+^-induced mitochondrial arrest (17).

If the molecular machinery that controls mitochondrial morphology has been well described in the last few years, more controversial is the relationship between mitochondrial shape and functionalities. Mitochondrial fusion, on the one hand, allows the exchange of material (mitochondrial DNA—mtDNA, proteins, lipids, metabolites) between separate regions of the network, allowing the repair of defective mitochondria and optimal utilization of the available substrates. Moreover, fusion is believed to dilute damaged molecules (such as mtDNA) and to protect mitochondria from engulfment within autophagosomes, upon nutrient depletion and autophagy induction (18). On the other hand, mitochondrial fission isolates dysfunctional mitochondria that are then directed along the autophagy route and eliminated (mitophagy), avoiding the accumulation of damaged mitochondria that may result in excessive ROS generation or inefficient ATP production (18). Moreover, the interplay between fission and fusion processes is also responsible for the dynamic nature of mitochondria in terms of cellular localization. In particular, fission allows the generation of sufficiently small mitochondria that can be easily transported along the cytoskeleton (19) and recruited to high energy-demanding regions within the cells. Finally, during cell cycle progression, mitochondrial morphology is continuously re-arranged. Fusion is predominant in G1/early S-phases, to favor mitochondrial respiration and sustain ATP production during cell growth (20). On the contrary, during S/G2/M phases, mitochondria are fragmented (to allow their equal distribution between daughter cells) and display a reduced respiration (to avoid DNA mutations during its synthesis, being mitochondrial respiration the major source of ROS) (21) (Figure 1A).

## Deregulated Mitochondrial Dynamics in Cancer Cells

Several defects in mitochondrial dynamics and positioning within the cell have been described in different cancer types and associated with development and progression of the disease. These alterations have multiple consequences in distinct physiological pathways controlling cell growth and survival, which have been also targeted by specific anticancer drugs. Notwithstanding, the precise mechanistic role of mitochondrial shape and organization in cancer progression is still unknown.

### Effects on Mitochondrial Metabolism

The reciprocal relationship between mitochondrial morphology and metabolism is poorly understood. In mammals, nutrients deprivation and starvation conditions induce mitochondrial fusion through a cAMP-mediated PKA activation and subsequent phosphorylation-dependent Drp1 inactivation (22, 23). Thanks to the elongated morphology, mitochondria can escape from engulfment within autophagosomes and degradation, likely because of a size-dependent steric hindrance. The induction of mitochondrial fusion and the protection from mitophagy provide starved cells with the advantage of preserving mitochondrial activity and sustaining ATP production. Indeed, not only a continuous mitochondrial matrix favors metabolites exchange and optimizes respiration (see below) but also elongated mitochondria are endowed with more cristae and display an increased dimerization/activity of the ATP synthase (22). On the contrary, fusion impairment causes ATP consumption and leads to starvation-induced cell death. On the same line, inactivation of pro-fusion proteins inhibits oxidative metabolism (24, 25), though the mechanism is not completely clear and may involve unbalanced partitioning of mtDNA. Galactose for glucose substitution enhances oxidative phosphorylation (OXPHOS), by markedly increasing mitochondrial fusion, expression of respiratory chain proteins and cristae density, without modifications in the overall mitochondrial mass (26). Moreover, the IMM-fusion protein Opa1 has been recently suggested to regulate the shape of cristae and favor the assembly/activity of the respiratory chain supercomplexes (27), further suggesting a link between mitochondrial morphology and activity. Finally, given that mitochondria have been suggested to supply membranes for autophagosome biogenesis (28), and autophagosomes have been demonstrated to originate from ER–mitochondria contact points (29), it is tempting to speculate that, during starvation, preserving mitochondria from degradation is essential to allow autophagy induction/progression.

It has been demonstrated that a continuous mitochondrial network favors the propagation within the matrix of Ca^2+^ waves. Indeed, upon its uptake, favored by the generation, on mitochondrial surface, of spatially and temporally restricted Ca^2+^ hot spots (30), the cation quickly spreads within the matrix. The Drp1-mediated mitochondrial fragmentation blocks this spreading, with important consequences on cell sensibility to Ca^2+^-mediated apoptosis (31). Given the importance of mitochondrial Ca^2+^ uptake not only for apoptosis but also for stimulation of the Kreb’s cycle and, ultimately, for mitochondrial respiration (32), it is tempting to speculate that an additional mechanism by which the morphology of mitochondria may regulate their activity is by affecting Ca^2+^ concentration within their matrix. Similarly, a continuous mitochondrial matrix, promoted by organelle fusion, likely enhances diffusion not only of Ca^2+^ but also of ADP, NADH, FADH_2_, and other metabolites (such as fatty acids) that fuel OXPHOS (33).

While mitochondrial fusion has been associated with increased OXPHOS, the contrary is generally true for fission, though the causative relationship between a reduced OXPHOS and fission events has not been completely clarified. For instance, hypoxia, a condition that dampens OXPHOS as a consequence of the limited amounts of available O_2_, has been reported to induce fission. The effect depends on the tuning of both Drp1 activity and Drp1–Fis1 interaction by the Siah2/AKAP121 complex (34, 35). Similarly, it has been recently reported that, upon electron transport chain inhibition, the energy-sensor enzyme AMPK quickly promotes mitochondrial fission, by directly phosphorylating and activating the OMM-located Drp1-receptor Mff (36). The physiological consequences of these fission events are not clear, though they could be linked to cell attempts to isolate and eliminate dysfunctional mitochondria through mitophagy (18). Overall, these observations suggest that mitochondrial metabolism is strictly associated with the regulation of mitochondrial fission/fusion machinery (37). In cancer, profound alterations in mitochondrial metabolism have been reported, but the relationship of these events with the observed changes in mitochondrial morphology (see below) is not clear. In particular, the so-called “Warburg effect,” consisting in an upregulation of glycolysis and in a limited utilization of pyruvate by mitochondria, is a common characteristic of many types of cancer. The expression of the recently identified mitochondrial pyruvate carrier, consisting of two distinct subunits, is downregulated in some cases (38). The fast, though poorly efficient, ATP production by glycolysis is believed to provide an advantage to rapidly dividing cells. Moreover, the upstream accumulation of glycolytic intermediates, due to the increased levels of cytosolic pyruvate, is useful for some anabolic processes. However, it is crucial to point out that, even upon conditions in which the Warburg effect has been activated, mitochondria keep their functionality, shifting their oxidative activity toward different substrates, such as glutamate and fatty acids [reviewed in Ref. (39)]. As an example, the entry of glutamate (derived from glutaminolysis) into the Kreb’s cycle (also known as tricarboxylic acid cycle, TCA) is important to sustain the levels of α-ketoglutarate, a critical intermediate in some biosynthetic pathways, such as that of non-essential-aminoacids. Cancer cells, characterized by a high-rate biosynthesis of different macromolecules, take advantage from a residual activity of the TCA cycle (40). Recently, in an elegant paper, it has been reported that the viability of several cancer cells depends on a constitutive, low-level ER-to-mitochondria Ca^2+^ transfer that sustains TCA cycle by enhancing the activity of three critical Ca^2+^-modulated enzymes (41). Again, beyond the Warburg effect, the maintenance of functional mitochondria and, in particular, of critical intermediates of the Kreb’s cycle, is necessary for cancer cell growth.

### Effects on Mitochondrial Ca^2+^ Signaling

The proper spatial distribution of the mitochondrial network plays a fundamental role in the maintenance of Ca^2+^ homeostasis. Indeed, mitochondrial Ca^2+^ uptake largely takes advantage of the close apposition of these organelles with the ER (42). The main Ca^2+^ releasing channels, the inositol 1,4,5-trisphosphate receptors, are present in ER membranes at sites of apposition with mitochondria, called mitochondria-associated membranes (43). Ca^2+^ release from these channels generates microdomains of high Ca^2+^ concentration at the ER–mitochondria interface. Ca^2+^ is rapidly taken up by mitochondria through the mitochondrial Ca^2+^ uniporter, quickly diffuses within the mitochondrial matrix through the network, and is extruded thanks to the mitochondrial Na^+^/Ca^2+^ exchanger or the Ca^2+^/H^+^ antiporter (32). As mentioned above, alterations in mitochondrial morphology can affect Ca^2+^ uptake as well as its spreading, with Drp1-mediated mitochondrial fission capable of limiting Ca^2+^ propagation and apoptosis induction (31). In particular, though it is current opinion that mitochondrial fragmentation is associated with apoptosis (see also below), a remarkable exception is represented by cancer cells. As an example, the myeloid cell leukemia factor 1 (Mcl-1), that belongs to the Bcl-2 family, exists in two different forms: the long splicing variant (Mcl-1L, anti-apoptotic) and the short one (Mcl-1S, pro-apoptotic). Mcl-1L is overexpressed in several malignancies (44); interestingly, it has been recently demonstrated that, by using splice-switching antisense oligonucleotides in different cancerous cells, lower Mcl-1L levels and higher Mcl-1S/Mcl-1L ratios induce mitochondrial hyperfusion (45, 46), due to a defective Drp1 translocation from the cytosol to mitochondria. This change in morphology has been associated with mitochondria hyperpolarization and, consequently, increased Ca^2+^ accumulation after exposure to specific stimuli that trigger apoptosis in a Ca^2+^-dependent manner, leading to increased sensitivity to cell death (45, 46). Thus, given the pivotal importance of mitochondrial Ca^2+^ handling in cancer cells proliferation and susceptibility to apoptosis [for a recent review, see Ref. (47)], and the impact of mitochondrial morphology on Ca^2+^ homeostasis, the fragmentation of the mitochondrial network often observed in tumor cells appears as an efficient mechanism to provide resistance to apoptosis (Figure 1B).

The relationship between mitochondria shape/transport along cytoskeleton and Ca^2+^ is bidirectional, due to the presence of a Ca^2+^-dependent regulation of these processes (48, 49) (see also above). In cancer, mutations in genes encoding proteins that control mitochondrial dynamics have been reported (see also below), such as those of adenomatous polyposis coli, recently identified as a Miro/Milton modulator (50). Though the relationship with Ca^2+^ homeostasis is not clear, given the presence of EF-hand domains in Miro1, the potential connection between deregulated Ca^2+^ signaling and alterations of mitochondrial transport/distribution in cancer appears as a promising area for future investigations.

### Effects on Cell Proliferation and Death

Several reports indicate that increased mitochondrial fission is necessary for proliferation and entry into S-phase, suggesting a link between mitochondrial dynamics and tumor progression (see also above). Moreover, multiple studies have reported a fragmented mitochondrial network in cancer [see Ref. (51) for a recent review] and the most frequently mutated signaling pathways in tumors, such as mitogen-activated protein kinase (MAPK), phosphoinositide 3-kinase (PI3K), and Myc pathways, have been consistently reported to directly impact on mitochondrial shape [reviewed in Ref. (52)]. In particular, altered levels (53) and/or cellular distribution (54) of Drp1 have been observed in tumor cells. An imbalance of Drp1/Mfn2 expression levels leading to mitochondrial fission has been showed in human lung cancer cell lines, as well as in lung tumor samples from patients. Restoration of a proper mitochondrial network in tumor cells, by Mfn2 overexpression, Drp1 inhibition, or Drp1 knockdown, resulted in a marked reduction of cell proliferation (53) and an increase in spontaneous apoptosis (55), dramatically decreasing the number of cancer cells in S-phase. Notably, restoring mitochondrial networking also reduced tumor progression in an *in vivo* xenotransplantation model (53). These observations suggest a key role for the mitochondrial network in modulating cell proliferation and apoptosis in cancer cells (Figure 1B). Intriguingly, mitochondrial network recovery is accompanied by organelle depolarization and increased oxidative stress, suggesting that a correct mitochondrial networking may induce cell cycle arrest, or spontaneous apoptosis, in cancer cells *via* modulation of mitochondrial ROS production (53). The early observation that cancer cells display higher ROS levels led to the conclusion that their targeting could be an efficient therapeutic strategy. Surprisingly, however, recent findings show that, in parallel to an increased ROS production, a strengthening of antioxidant defense may favor tumor survival. These results suggest that ROS are, on the one hand, crucial for cell signaling and proliferation, enhancing cell division, but, on the other, have to be finely tuned, avoiding an excessive ROS accumulation that induces cytotoxic effects. It is thus tempting to speculate that keeping ROS level within a precise window is necessary for tumor progression [reviewed in Ref. (39)]. As an example, H_2_O_2_ oxidizes and blocks the activity of the tumor suppressor PTEN (56), but the cytotoxicity induced by high levels of ROS leads several tumors to upregulate antioxidant pathways.

The well-known positive correlation between apoptosis and mitochondrial fission appears counterintuitive in cancer cells that actively evade cell death. As seen above, mitochondrial fragmentation in cancer may be a possible mechanism to escape Ca^2+^-dependent apoptosis. In addition, it has been reported that an excessive mitochondrial fragmentation, such as that observed upon Mfn1 ablation, induces a curvature of the OMM, which is not compatible with Bax association, thus causing resistance to apoptosis (57). Given that Mfn1 depletion has been associated with invasive cancer types (58), it seems reasonable to hypothesize that tumors may have developed the ability to resist to apoptosis, despite their frequently fragmented mitochondrial morphology. Furthermore, a relationship between cancer progression and changes in the relative expression levels of both the pro-apoptotic and anti-apoptotic members of the Bcl-2 family has been established [reviewed in Ref. (59)]. Importantly, several of these proteins have been reported to actively modulate mitochondrial morphology, with a direct impact on the modulation of apoptosis [reviewed in Ref. (60)]. For instance, loss of the pro-apoptotic protein Bax has been described in certain type of tumors (61), and Bax/Bak deficiency is associated with mitochondrial fission and resistance to apoptosis (62). In cancer, mitochondrial fragmentation not only favors resistance to apoptosis, but it has been additionally reported to promote cell proliferation. As an example, upregulation of the MAPK pathway in several tumors activates Drp1 by an ERK-mediated phosphorylation at serine 616 (63, 64), promoting mitochondrial fission. Importantly, Drp1 expression is necessary for RAS-mediated oncogenic transformation (64). More controversial is the role of Fis1 in cancer. Fis1 downregulation, while inhibiting mitochondrial fission, has been reported to reduce apoptosis by dampening Bax translocation to the OMM (65). Accordingly, Fis1 overexpression promotes apoptosis (7) and the effect has been suggested to depend on ER-to-mitochondria Ca^2+^ transfer (66). Specifically, Fis1 interacts with Bap31 localized at the ER, facilitating its cleavage into the pro-apoptotic p20Bap31 and building a platform for procaspase-8 recruitment and activation (67). The pro-apoptotic Fis1 activity, however, is likely independent from its fission activity, since it is conserved when mitochondrial morphology is corrected by manipulating Opa1 level (65). In adrenocortical cancer cells, the microRNA miR-484 suppresses Fis1 translation and inhibits apoptosis (68), while miR-483-5p targets Fis1, inhibits mitochondrial fission and cisplatin sensitivity in tongue squamous cell carcinoma (69). Overexpression of Fis1 (and of Drp1) has been observed in oncocytic thyroid tumors (70) and has been associated with poor prognosis in patients with acute myeloid leukemia (71). Thus, either positive or negative correlation between Fis1 expression and tumorigenesis has been observed, though the former is predominant. Importantly, again, it seems reasonable to hypothesize that this is independent from the Fis1 pro-apoptotic activity and likely linked to its pro-fission effects.

Finally, mitochondrial dynamics seems to play a role in another important cancer signaling pathway. Organ size, in flies as well as vertebrates, is controlled by the Hippo pathway, which directly promotes cell proliferation and represses apoptosis [reviewed in Ref. (72)]. Interestingly, the mitochondrial network is also an important target of this pathway, as its activation in flies causes an increase in mitochondrial fusion, due to direct transcriptional regulation of major mitochondrial fusion genes. Importantly, genetic reduction of mitochondrial fusion suppresses Hippo pathway-dependent cell proliferation and tissue overgrowth, sustaining the importance of the connection between this signaling route and mitochondrial dynamics in both normal development and cancer biology (73).

### Effects on mtDNA Exchange and Mitophagy

One of the consequences of the dynamic nature of the mitochondrial network is the exchange of mtDNA that allows mitochondria to keep their integrity, ensuring a healthy organelle population, thus protecting the cell from detrimental effects of (accumulating) mtDNA mutations. Different studies have suggested a role for germline mtDNA mutations in the development of a wide variety of cancers [reviewed in Ref. (74)]. In particular, it has been suggested that fusion may promote respiratory efficiency by complementation of mtDNA mutations (18). One can hypothesize that somatic mtDNA mutations, not corrected because of defects in the fission/fusion machinery, can be selected and expanded, resulting, for example, in a predominantly glycolytic metabolism that favors tumor progression (Figure 1B). Despite this evidence, the hypothesis that somatic mtDNA mutations expand within the mitochondria population, leading to cancer development, has not been clearly demonstrated yet. Moreover, increased fusion has been shown to promote OXPHOS quite rapidly (26), suggesting that the effects of fusion on oxidative metabolism are post-translational and not due to mtDNA complementation.

Regarding mitophagy, its role in tumorigenesis is debated. While Parkin ablation (that inhibits mitophagy) has been associated with enhanced cancerous transformation (75), upregulated mitophagy has been observed upon K-Ras-driven oncogenic transformation (76). Likely, the impact of mitophagy on cancer progression may depend on the specific stage of the tumor and be related to the need for cancer cells to establish a precise window of ROS levels [see above and reviewed in Ref. (39)]. Similarly, a dichotomous activity of the mitochondrial biogenesis regulator PGC-1α in cancer viability has been reported [reviewed in Ref. (39)], further suggesting that mitochondrial dynamics are actively modulated during tumorigenesis.

## Mitochondria Cell Positioning and Cancer Cell Migration

Increased perinuclear localization of mitochondria has been reported under various conditions such as hypoxia, mitophagy, and early phases of ER stress (77). To what extent the control of mitochondrial dynamics, in terms of changes in mitochondrial subcellular spatial organization, is deregulated in cancer has been less frequently assessed. Only recently, a number of studies have correlated the subcellular distribution of mitochondria with the migration and invasion of cancerous cells. The issue of cell migration is of significant importance since cancer metastasis is the major cause of tumor-associated death. For this reason, cancer research has been focused on the identification of regulatory mechanisms controlling active cell migration. In general, mitochondrial fission has been shown to be necessary for the invasion potential of thyroid, breast, and glioblastoma cancer cells [reviewed in Ref. (52)]. Different mechanisms have been proposed for the regulation of fission/fusion machinery in cancerogenesis and metastasis propagation. As an example, nuclear factor κB (NF-κB)-inducing kinase (NIK), a constitutively active kinase localized in the OMM and stimulating non-canonical NF-κB signaling pathways, has been implicated in tumorigenesis and metastasis (78). Recently, Jung et al. discovered a novel function for NIK in regulating mitochondrial dynamics in cultured glioma cells. They showed that NIK promotes mitochondrial fission in a Drp1-dependent manner, resulting in the translocation of mitochondria to the leading edge of cancer cells and stimulating their invasive behavior independent of NF-κB signaling (79). In another recent study, PI3Ks, master regulators of cellular metabolism commonly altered in cancer, have been linked to mitochondria remodeling. In particular, it has been demonstrated that cancer cells exposed to PI3K antagonists triggered the transport of energetically active, elongated mitochondria to the cortical cytoskeleton of tumor cells. In turn, these repositioned mitochondria supported increased lamellipodia dynamics, faster turnover of focal adhesion complexes, heightened velocity and distance of random cell migration, and increased cancer cell invasion. Importantly, tumor invasion is suppressed when Mfn1 is silenced, further supporting a role of mitochondrial fission/fusion dynamics in cancer cell migration (80). Of note, despite PI3K and downstream kinases are amenable to pharmacological interventions, making this pathway an attractive target, drug resistance and tumor adaptation mechanisms hampered the exploitation of this therapeutic strategy, suggesting that combination of PI3K inhibitors with compounds that disable mitochondrial reprogramming would likely provide more effective anticancer regimens (81). Finally, Howe and colleagues have recently demonstrated that mitochondria rapidly traffic into leading edge structures during cell migration, a process driven by AMPK. The authors propose that cell migration demands localized energy supply to fuel the leading edge migration machinery. The restricted high ATP consumption promotes local activation of AMPK, which in turn stimulates directional microtubule-based trafficking of mitochondria to produce and replenish ATP. Leading edge mitochondria were smaller and less networked than those located in the cell body, suggesting the concerted action of the mitochondria fission/fusion and motility machineries to regulate cell migration (82) (Figure 1C).

## Pharmacological Perspectives and Conclusion

Many of the reported findings suggest that the pattern of mitochondria dynamics and positioning can be specific to different cancer types. Thus, profiling mitochondrial phenotype in healthy and tumor cells could ultimately allow to define organelle outlines that correlate with mitochondrial metabolic state and provide a non-invasive biomarker for cancer diagnosis. Indeed, the shape of the mitochondrial network has already been proposed as a diagnostic tool to distinguish healthy from cancerous tissue. In normal human skin, a very distinct pattern of mitochondria that changes as a function of depth is present, while in cancer patients, this organization is basically abolished. This allows non-invasive microscopy techniques to assay mitochondria shape and arrangement in human tissues (83). Recently, a new technique that exploits NADH autofluorescence used lasers to take optical sections of mitochondria naturally “stained” by NADH. An automated method to assess mitochondria organization *in vivo* in each layer of skin tissue has also been developed (84). Interestingly, mitochondrial patterns can also be used as biomarkers of drug response, verifying the efficacy of antiproliferative therapeutic interventions.

Other biomarkers have been proposed to be used for cancer early detection and therapy. For instance, total Drp1 levels and Drp1 phosphorylation on Ser616 have been demonstrated to positively correlate with breast cancer and melanoma progression and with ERK phosphorylation/oncogenic MAPK signaling (63, 64, 85). Accordingly, compounds or peptides able to inhibit Drp1 activity have been tested and demonstrated to slow down the growth of certain tumors (52, 86). Pharmacological and genetic Drp1 inactivation inhibit glioblastoma growth (87), as well as hepatocellular carcinoma cell proliferation (88). Interestingly, high mitochondrial lacunarity (linked to mitochondrial fragmentation) correlates with increased sensitivity to the mitochondrial fission inhibitor Mdivi-1 of mesothelioma cells (89).

Altogether, these observations indicate that there is a strict relationship between mitochondrial dynamics and cancer progression. In particular, impaired fusion and enhanced fission seem to contribute to the proliferation/apoptosis imbalance in cancer. Thus, a mitochondrial-targeted strategy for cancer therapy, i.e., targeting mitochondrial networking by modulating proteins involved in determining mitochondrial morphology, could represent an intriguing complementary approach to other emerging treatments (such as those targeting mitochondrial metabolism) for an efficient cancer therapy.

## Author Contributions

All authors listed have made substantial, direct, and intellectual contribution to the work and approved it for publication.

## Conflict of Interest Statement

The authors declare that the research was conducted in the absence of any commercial or financial relationships that could be construed as a potential conflict of interest. The reviewer, GM, and handling editor declared their shared affiliation, and the handling editor states that the process nevertheless met the standards of a fair and objective review.
